# Social bees are fitter in more biodiverse environments

**DOI:** 10.1038/s41598-018-30126-0

**Published:** 2018-08-17

**Authors:** Benjamin F. Kaluza, Helen M. Wallace, Tim A. Heard, Vanessa Minden, Alexandra Klein, Sara D. Leonhardt

**Affiliations:** 1 Department of Ecology, Leuphana University, 21335 Lüneburg, Germany; 2 Genecology Research Centre, Faculty of Science, Health, Education and Engineering, University of the Sunshine Coast, Maroochydore, 4558 Australia; 3 CSIRO Ecosystem Sciences, Brisbane, 4001 Queensland Australia; 4 Institute of Biology and Environmental Sciences, University of Oldenburg, 26111 Oldenburg, Germany; 5 Department of Biology, Ecology and Evolution, Vrije Universiteit Brussel, 1050 Brussels, Belgium; 6 Department of Nature Conservation and Landscape Ecology, University of Freiburg, 79085 Freiburg, Germany; 7 Department of Animal Ecology and Tropical Biology, University of Würzburg, 97074 Würzburg, Germany; 8 Present Address: School of Life and Environmental Sciences, The University of Sydney, NSW, 2006 Australia

## Abstract

Bee population declines are often linked to human impacts, especially habitat and biodiversity loss, but empirical evidence is lacking. To clarify the link between biodiversity loss and bee decline, we examined how floral diversity affects (reproductive) fitness and population growth of a social stingless bee. For the first time, we related available resource diversity and abundance to resource (quality and quantity) intake and colony reproduction, over more than two years. Our results reveal plant diversity as key driver of bee fitness. Social bee colonies were fitter and their populations grew faster in more florally diverse environments due to a continuous supply of food resources. Colonies responded to high plant diversity with increased resource intake and colony food stores. Our findings thus point to biodiversity loss as main reason for the observed bee decline.

## Introduction

Ongoing pollinator declines threaten spatial and temporal stability of pollination and thus global food production^1–5^. Natural habitat loss and intensive land use have been repeatedly highlighted as key drivers of pollinator population declines, but empirical evidence for their impact on specific pollinator species is lacking^4,6–10^. Bees, in particular the highly social species, are among the most important pollinators globally^2^, and spatial and temporal stability of pollination is strongly correlated with bee abundance and diversity^11,12^. Bees in turn depend entirely on flowering plants for food^13^, and higher bee abundances are typically correlated with higher plant diversity and thus food source diversity^8,14,15^. These correlations do however not show whether more biodiverse habitats actually increase bee populations (e.g. through enhancing reproductive fitness) or simply attract more foragers to rewarding food patches^16^. In fact, it is unknown whether changes in biodiversity, and thus food source diversity and abundance, actually impact social bee population dynamics. If we want to fully grasp the intricate relationship between biodiversity and stable bee populations (and thus ultimately food production)^17^, we need to clarify how the diversity of food sources drives the fitness and thus population dynamics of bees^18^.

Surprisingly, only a handful of studies have examined bee fitness in relation to flowering plant diversity^19–25^. They all revealed that floral diversity positively affected colony growth or offspring production. However, these studies were confined to bee species with seasonal life cycles, i.e. solitary bee species and (primitively eusocial) bumble bees. In fact, bumble bees and solitary bees may be more severely affected by reduced floral diversity than the highly social bees (i.e. honeybees and stingless bees) which typically store floral resources over prolonged periods^26,27^ and may thus better survive resource shortages. However, for the globally distributed and often managed highly social bees, we still lack longer term data on colony reproduction or population growth in relation to food source diversity, abundance *and* nutritional quality. In this group, fitness and population growth are notoriously hard to quantify due to management bias (e.g. supplementary feeding) and long-lived colonies. Moreover, while some more recent studies have related floral resource diversity and/or availability to brood production and overwintering survival in *Apis mellifera*^25,28–30^, equivalent studies are entirely lacking for stingless bees (Apidae: Meliponini).

To understand the impact of biodiversity on bee fitness and population growth, we examined how floral resource diversity, abundance *and* nutritional quality affected stingless bees, i.e. highly social bees with perennial life cycles. We hypothesized that (i) increasing plant diversity (i.e. plant species richness) would strongly increase (reproductive) fitness and thus colony population growth and that (ii) plant species richness would better explain observed fitness effects than habitat type. Further, we predicted that (iii) fitness and colony population growth would be affected by both the quantity and the nutritional quality of food stored by colonies.

## Material and Methods

Our study was conducted from 2011 to early 2014 in South East Queensland, Australia (24°38′–27°30′ S, 152°6′–153°7′ E) and used the Australian stingless bee *Tetragonula carbonaria* Smith as our model organism^31^. It covered three habitat types with different plant diversity levels (agricultural areas (i.e. macadamia plantations), natural forests and gardens). At each of these sites, we measured plant diversity by assessing plant species richness. Plant species richness was generally lowest in plantations and highest in gardens, and correlated with collected resource diversity^32^. Plant species richness further correlated with the area of all three habitat types (see Supplementary Information SM 1: Supplementary Table S1a), which is why we analyzed plant species richness and habitat area separately. We considered fitness in the sense of reproductive fitness and measured the reproductive output of a colony, which is generally considered the reproductive entity in highly social animals^33^.

### Experimental setup

Bee colonies were placed along the gradient of varying plant species and thus resource diversity and abundance covering the three habitat types. Two *T*. *carbonaria* colonies (in hives) were placed at each of eight paired sites (replicates) per habitat type with a minimum distance of 55 m in between, creating a nested design of 24 paired sites. In gardens, distances between paired sites were greater (706 ± 129 m) because of limited suitable sites. Due to early usurpation by another bee species, two colonies were excluded, resulting in 46 original bee hives. In plantations, bee hives were closed for 24 h to prevent contamination from insecticides during pest control. Note that we had observed *T*. *carbonaria* foragers of naturally occurring nests in all habitats (except for plantations), before introducing colonies and starting our experiment.

Botanical surveys were conducted along four 500 m transects in four directions (north, south, east and west, each 5 m wide) for each study location (i.e. two paired sites) to assess plant species richness and resource abundance. Because of the greater distances between paired sites in gardens, we conducted separate botanical surveys for both paired sites, resulting in 8 plant species richness and resource abundance measures (and thus eight data points) for gardens. Plant form (categories: herb, shrub or tree) and abundance (rare, uncommon or common) of each plant species was recorded (see^32^ for details). The relative frequency for each combined category (plant form × abundance; e.g. rare herb) was calculated per site and multiplied by a weighting factor (obtained through model optimization for explaining variance in flight activity, see^32^). The sum of all combined category values resulted in the plant resource abundance value for each site. As we had no information on the resource plants used by stingless bees, we considered all flowering plants to provide some sort of resource, either pollen or nectar.

We quantified the proportional area of each habitat type (plantation, forest, garden) for each site within the bee’s flight radius. *T*. *carbonaria* are known to forage within a flight radius of 500 m of the hive^34^. We therefore classified all habitats within a 500 m radius of the hive using aerial photos obtained by Google Earth (software: KML Toolbox, Zonum Solutions, 2012). Sites were attributed to one of the three habitat types if its area accounted for >75% of the total habitat area within flight range. The target habitat accounted for on average 90% for plantation (with the rest habitat being forest), 90% for forest (rest being fields and gardens) and 82% for garden (rest being water). All habitat patches identified for each site were validated by ground surveys^31^.

To quantify (reproductive) fitness, we recorded five parameters for individual bee (i.e. worker size and body fat) and colony performance (i.e. brood size, daily worker production, number of queen pupae) as well as colony reproduction and thus colony population growth.

### Colony reproduction and population growth

**Figure 1:**
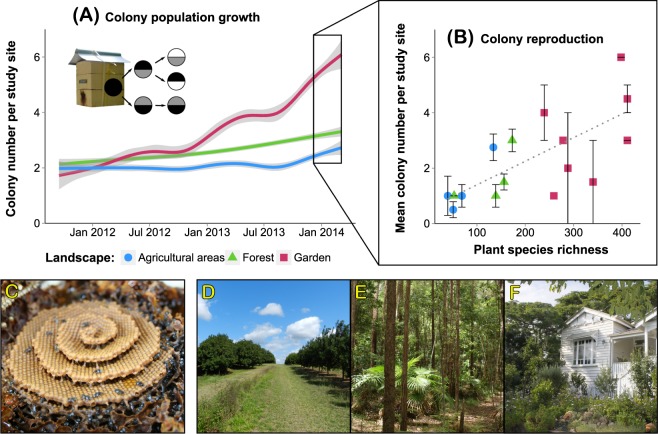
(**A**) Colony population growth (change in number of colonies per study site over two years) and (**B**) colony reproduction (mean colony number per site in March 2014 with standard errors) in relation to plant species richness within the bees’ flight radius (i.e. 785,000 m^2^) in different habitats (i.e. Landscape: agricultural areas: *blue circles*, natural forests: *green triangles*, suburban gardens: *red squares*). (**A**) Colonies were propagated by splitting the brood (*full circle*) and equipping each half with new boxes (step 1: *grey semi-circles*); splits were repeated when brood had regrown (step 2, adding new boxes: *white semi-circles*). Colored lines denote changes in average colony numbers per habitat type over two years including standard errors (*grey margins*). (**B**) The original 46 mother colonies installed at sites in 2011 were propagated into a total of 93 daughter colonies by March 2014 (mean ± standard deviation; agricultural areas: 3 ± 2 per site; forests: 3 ± 2; gardens: 6 ± 4). Colony population growth was best explained by overall plant species richness in the surrounding habitat (Supplementary Table S4; GLMM: *χ*^2^ = 15.03, *df* = 1, *P* < 0.001). The number of daughter colonies produced by a mother colony within 2 years significantly increased with increasing plant species richness (Spearman correlation test: *r* = 0.59, *P* < 0.001, *dotted line*), and was highest in gardens and lower in forests and agricultural areas (Tukey test: agricultural areas vs. forests: *P* = 0.783; forest vs. gardens: *P* = 0.039; agricultural areas vs. gardens: *P* = 0.007). Photos: Brood spiral of *Tetragonula carbonaria* colony (**C**), macadamia plantation (**D**), natural forest (**E**) and suburban garden (**F**). Note that the greater number of garden sites was due to the greater distance between paired sites which necessitated separate botanical surveys for each site (**B**).

Colonies of *T*. *carbonaria* were kept in hives (consisting of two boxes housing the brood and an additional box used as honey super) and artificially propagated to measure their reproductive output following Heard^35^. For most stingless bees, colony growth is limited by nesting space under natural conditions^27,36^. We thus provided unlimited nesting space by performing hive splits (hive: colony + wooden box:^35^) to separate resource effects from nesting space limitations. When a hive was split, brood and food storage were separated by a horizontal cut between the centre and bottom box and both hive parts were then equipped with new empty boxes (either one empty bottom box, or two: centre and super box; Fig. 1A insert). As a new queen is raised in each hive part^35^, a hive split effectively creates two daughter colonies of the same lineage from one mother colony. We always kept the daughter colony at the original location of the mother colony.

Hives were always split when they reached a total weight of 8.5 kg (weight empty box: 4.7 kg ± 0.6), equivalent to approximately 70% nest space used and thus close to when the colony would initiate natural colony fission. Thus, the number of colonies descending from one mother colony corresponded to its reproductive output. The increase in the number of colonies per site over time in turn corresponded to colony population growth.

### Performance

#### Colony performance

Each bee colony was opened once per year in 2012 and 2013 to record colony performance and to obtain samples of pollen, honey and adult bees. At least one colony was opened at each paired site (1–4) in each season (wet, cold and dry; total of 35 in forests, 42 in gardens, 33 in plantations).

Nests of *T*. *carbonaria* consist of a circular brood in shape of an upright elongated sphere (ellipsoid) surrounded by honey and pollen storage pots (Fig. 1C). The brood is arranged in a spiral which perpetually grows upwards when new brood cells are built^37,38^. This advancing front of the brood continuously fills the empty space successively freed by hatching pupae on top. All open worker cells at the advancing front form one batch and are synchronously built and provisioned. Thus, batch size corresponds to the number of workers produced per day^39^. Queens are continuously produced and queen pupae are easily identified by their larger size and location at the rim of the brood layers^39^.

We assessed colony performance by recording a) number of open worker cells in the currently provisioned batch (worker production), (b) number of queen pupae at the lowest brood layers with pupae above the advancing front (queen production) and c) total brood volume. Worker and queen production could be recorded for 56% of our colonies (when locating the advancing front). Brood volume was obtained for all opened colonies by measuring the width (*w*) and length (*l*) of the largest brood layer as well as the depth of the brood comb in the top (*d*_*t*_) and bottom (*d*_*b*_) box. Depth was measured by piercing the center of both brood hemispheres with a long glass pipette. The total brood volume (*V*) was then calculated using the formula for an ellipsoid:

1 $$V=\frac{4}{3}\pi \,\ast \,\frac{w}{2}\,\ast \,\frac{l}{2}\,\ast \,\frac{({d}_{t}+{d}_{b})}{2}$$

Brood volume was highly correlated with brood circumference (see SM 1).

#### Individual bee performance

We assessed the fitness of individual workers based on body size and body fat, which positively correlate with the feeding status and/or survival of insects in general and bees in particular^40–43^.

Bee body fat was measured using the protocol of Cook, Eubanks, Gold and Behmer^41^. Before colonies were opened to obtain samples (see above), we captured departing adult bees by placing a clean clear plastic bag over the entrance hole. Captured bees were killed by freezing and dried for 24 h at 50 °C to evaporate water and melt wax residuals. We then pooled 15 individuals per colony and weighed and extracted the bulk sample in chloroform for 24 h. Chloroform and dissolved lipids were removed and discarded. The procedure was repeated three times and remaining chloroform was evaporated in a heating cabinet for 48 h at 30 °C. The bulk sample was finally weighed again to determine weight loss (equivalent to the weight of lipids extracted from bees).

Bee body size was assessed in November 2012 for adult bees caught within a single season. Ten bees per colony (for 13 colonies in plantations, 15 in forests and 12 in gardens) were dissected under a stereo microscope (Kyowa model SZM, Kyowa Optical Co. Ltd, Sagamihara, Japan) and individual body parts were mounted on clay. Head length and width, mesonotum length and width, upper and lower interocular distance and intertegular distance were measured (in mm) as biometric parameters^40,44,45^. A principal component analysis (PCA) was performed on all biometric parameters and the first axis (explaining 61% of the variance across samples) was used as body size parameter in subsequent statistical analyses.

### Food quantity and nutritional quality

*Tetragonula carbonaria* stores honey and pollen in separate pots^37^. We collected honey and pollen samples from 1–10 pots of varying age from each colony to determine food nutritional quality. Honey samples were analyzed for their sucrose and water content using hand-held refractometers (sucrose: Eclipse Refractometer, Bellingham + Stanley Ltd., Lawrenceville, USA; water: HHR-2N Honey Refractometer, ATAGO Co. Ltd., Tokyo, Japan) and for acidity using standard pH-test strips. Pollen samples were analyzed for their amino acid composition and total protein (i.e. sum of all amino acids) content as well as for their elementary composition (see Supplementary Information SM 2 for analytical details). A PCA was performed on all single amino acids and the first axis (explaining 80% of the variance across samples) used as parameter for pollen amino acid content in further statistical analysis (Supplementary Table S2). We additionally used total protein (mg/g) and the sum of all essential amino acids (mg/g) as response variables in statistical analyses (Supplementary Table S2), because bees appear to be primarily affected by overall protein content rather than amino acid composition of pollen (reviewed by^46^). Likewise, a principal component analysis was performed on all micro-elements, and the first axis of the PCA (explaining 87% of the variance across samples) was used for further analyses. The macro-elements phosphorus, nitrogen and carbon were entered as separate factors in the correlation analyses (see Supplementary Information SM 1: Table S1a,b).

### Statistical details

We composed a correlation matrix to identify correlations between all recorded variables, for explanatory variables (related to a) plant diversity and b) stored food quantity and nutritional quality) and response variables (related to c) colony and individual bee performance and d) colony reproduction and thus population growth; see Supplementary Information SM 1). Because plant species richness was positively correlated with garden area, and negatively correlated with forest and agricultural area (see Supplementary Information SM 1: Table S1a), we performed separate models for plant species richness and habitat areas to determine which variable provided more explanatory power in describing fitness (see Supplementary Information SM 3). Plant species richness explained the observed variance best (SM 3) and was therefore tested in all subsequent analyses.

We used generalized linear mixed effect models (GLMM) to analyze the effect of fixed explanatory variables on fitness (i.e. colony population growth, brood volume, queen production, and worker production) and individual bee performance (i.e. worker body fat and worker body size). Because of co-variation between explanatory variables (see Supplementary Information SM 1), we performed separate analyses for plant diversity and resource related variables (termed biodiversity and resource model, see Supplementary Table S4). We first tested the effect of plant diversity-related explanatory variables (biodiversity model, i.e. habitat types, plant species richness and resource abundance) on fitness response variables. We then tested the effect of non-correlated explanatory variables related to stored food quantity and nutritional quality (resource model, i.e. weight of pollen and honey storage, total protein in pollen and sucrose concentration in honey) on the same fitness variables recorded for the same day (i.e. brood volume, queen production, worker production and worker body fat).

We always started with the most complex model which included all explanatory variables and their interactions, followed by step-wise simplification of models by excluding interactions and variables as suggested by Zuur *et al*.^47^. Model quality was evaluated using Akaike’s Information Criterion (AIC), and the model with the lowest AIC value was considered the model with the highest explanatory value. Model selection was further confirmed by testing whether individual explanatory variables (remaining in the most parsimonious models) explained a significant proportion of the overall variance by comparing the model with a given explanatory variable to the same model without this variable (anova command in the lme4 package which compares two nested models using REML scores^48^). In order to compare effects of different explanatory variables on specific response variables, the explained variance (*R²*) of the best model following AIC selection was calculated as described by Nakagawa and Schielzeth^49^, and compared between models (library MuMIn^50^).

Colony reproduction and queen production were entered in GLMMs using a Poisson distribution. All other response variables (brood volume, worker production, worker body fat and size) were analyzed by GLMMs with Gaussian distribution. To take into account slight overdispersion of models on queen production (i.e. 2.0), we only considered p-values below 0.01 significant, as overdispersion can inflate significance levels. Variables were square root transformed where necessary (brood volume) to achieve normality. Colony/hive nested within site was entered as a random effect in all models (with the exception of colony reproduction: only site entered as a random effect) to account for repeated sampling of the same colony and the nested study design. Year was included as additional random factor in models for brood volume, worker production and worker body fat. Differences between habitat types were assessed using Tukey’s HSD post hoc test (package multcomp^51^), and effects of plant richness were calculated using Spearman correlation tests. All analyses were performed in R (v.2.15.0).

### Data availability

Data on recorded fitness parameters in relation to habitat and plant species richness is available at research gate: doi: 10.13140/RG.2.2.35797.73447.

## Results

### Colony fitness

The original 46 mother hives as installed at sites in 2011 were propagated into a total of 93 bee hives by March 2014 (mean ± standard deviation; plantations: 3 ± 2 per site; forests: 3 ± 2; gardens: 6 ± 4; Fig. 1). Total hive reproduction was best explained by overall plant species richness in the surrounding habitat (biodiversity model, Supplementary Table S4; GLMM: *χ*^2^ = 15.03, *df* = 1, *P* < 0.001). The number of hives produced by a mother colony within 2 years significantly increased with increasing plant species richness (correlation test: *r* = 0.59, *P* < 0.001), and was highest in gardens and lower in forests and agricultural areas (Tukey test: agricultural areas vs. forests: *P* = 0.783; forest vs. gardens: *P* = 0.039; agricultural areas vs. gardens: *P* = 0.007).

**Figure 2:**
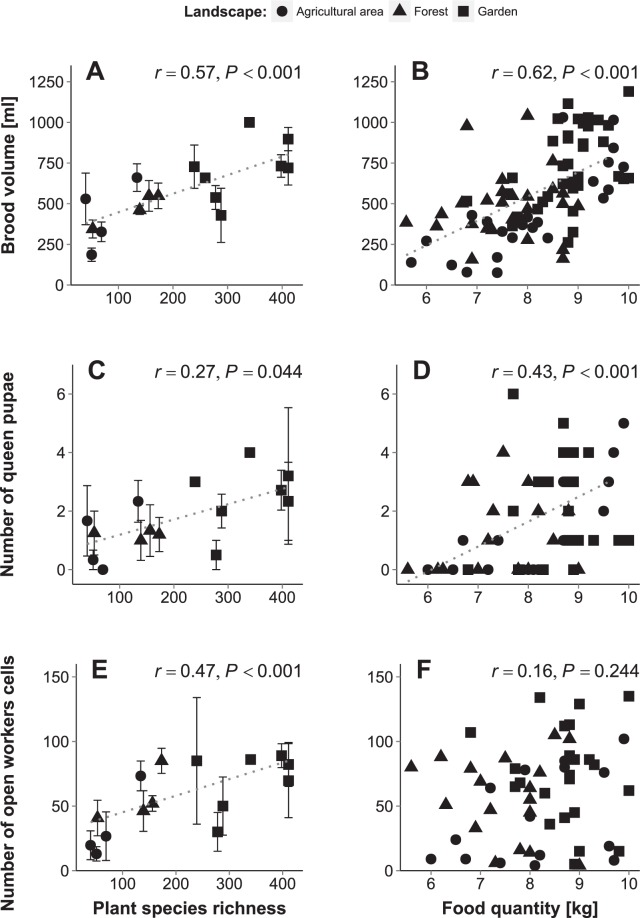
Bee colony fitness parameters in relation to plant species richness within the bees’ flight radius (**A**,**C**,**E**) and stored food quantity (**B**,**D**,**F**) in different habitats (Landscape: agricultural areas (*circles*), forests (*triangles*) and gardens (*squares*)). Dotted lines indicate significant correlations (Spearman). Points in A,C and E display means and standard errors where several measurements could be taken of different colonies at a specific site. Both brood volume and queen production (i.e. number of queen pupae) of bee hives were best explained by plant species richness (Supplementary Table S4; brood volume: GLMM: *χ*^2^ = 20.88, *df* = 1, *P* < 0.001; queen production: *χ*^2^ = 6.82, *df* = 1, *P* = 0.009) and increased with plant species richness (**A**,**C**). Brood volume and queen production were also better explained (or tended to better explained) by stored food quantity than nutritional quality (Supplementary Table S4; brood volume: *χ*^2^ = 18.79, *df* = 1, *P* < 0.001; queen production: *χ*^2^ = 5.32, *df* = 1, *P* = 0.021) and increased with increasing stored food quantity (**B**,**D**). Daily worker production (i.e. number of open worker cells) was best explained by plant species richness interacting with habitat type (Supplementary Table S3; habitat: *χ*^2^ = 9.64, *df* = 4, *P* = 0.047, plant species richness: *χ*^*2*^ = 13.61, *df* = 3, *P* = 0.003, **E**). It generally increased with plant species richness (**E**), particularly in agricultural areas (*r* = 0.68, *P* = 0.005) and forests (*r* = 0.51, *P* = 0.03), but not in gardens (*r* = 0.19, *P* = 0.379). Interestingly, worker production was not affected by stored food quantity and nutritional quality, but sites and year (Supplementary Table S3; **F**). Note that the greater number of garden sites was due to the greater distance between paired sites which necessitated separate botanical surveys for each site.

Both brood volume and queen production (i.e. number of queen pupae) of bee hives were also best explained by plant species richness (Supplementary Table S4; brood volume: GLMM: *χ*^2^ = 20.88, *df* = 1, *P* < 0.001; queen production: *χ*^2^ = 6.82, *df* = 1, *P* = 0.009) and likewise increased with plant species richness (brood volume: Fig. 2A, correlation test: *r* = 0.54, *P* < 0.001; queen production: Fig. 2C, *r* = 0.30, *P* = 0.002). When testing for effects of stored food quantity and nutritional quality, brood volume and queen production were (tentatively) best explained by and increased with food storage weight (resource model, Supplementary Table S4; brood volume: GLMM: *χ*^2^ = 18.79, *df* = 1, *P* < 0.001, Fig. 2B, correlation test: *r* = 0.63, *P* < 0.001; queen production: *χ*^2^ = 5.32, *df* = 1, *P* = 0.021, Fig. 2D, *r* = 0.49, *P* < 0.001).

Worker production (i.e. number of open worker cells per batch) was best explained by plant species richness interacting with habitat (Supplementary Table S4; landscape: GLMM: *χ*^2^ = 9.64, *df* = 4, *P* = 0.047; plant species richness: GLMM: *χ*^2^ = 13.61, *df* = 3, *P* = 0.003, Fig. 2E). The number of worker cells increased with plant species richness in agricultural areas (correlation test: *r* = 0.68, *P* = 0.005) and forests (*r* = 0.51, *P* = 0.03), but not in gardens (*r* = 0.19, *P* = 0.379). However, when testing for the effect of stored food quantity and nutritional quality, the NULL-model (i.e. only random effects of sites and year) best explained observed variance (Supplementary Table S4).

### Individual fitness

Fitness of individual workers, i.e. worker body fat and size, showed overall little variance across all observations and was best explained by random effects (i.e. NULL-models) in biodiversity models (Supplementary Table S4). Worker body fat was also best explained by the NULL-model when testing stored food quantity and quality variables (data not available for body size as body size was measured separately from food quantity and quality and could therefore not be related).

## Discussion

Our study demonstrates that fitness and population growth of highly social bees is best explained by and positively correlated with plant diversity (Figs 1 and 2). The positive effect of plant diversity was most likely driven by continuous resource availability and thus greater food quantity over time (Fig. 2, Supplementary Table S4). For example, foraging activity as well as pollen and nectar intake were generally higher at plant species rich (e.g. garden and forest) sites than at plant species poor agricultural sites, and variation between the Australian wet and dry season (where temperatures are typically sufficiently high to induce regular foraging) was least pronounced for garden sites^31^, which indicates a continuous resource allocation. In contrast to other studies suggesting that the size of natural habitats explains bee population dynamics^52–55^, we found that overall plant species richness was a significantly better predictor of fitness and population growth (Supplementary Table S4). Moreover, in a related study, we demonstrated that these generalist social bees also collected more diverse pollen in more diverse habitats^32^. Higher plant species richness in the surrounding habitat was further positively correlated with a higher number of plant species in the larval provisions produced by their colonies (as assessed by metabarcoding, M Trinkl, A Keller & SD Leonhardt, unpublished data). The relationship between high plant species richness and thus available resource diversity, high pollen diversity and quantity intake and high source richness of produced larval provisions strongly indicates a direct link between resource diversity and fitness benefits. Moreover, colonies were even able to thrive and reproduce in an agricultural plantation where they had access to a habitat patch of high plant species richness (e.g. a small remnant of natural vegetation) which increased overall plant species richness beyond the level normally found for this habitat and comparable to forest sites (see plantation site with a plant species richness of 134 species as assessed by transect walks: Figs 1B and 2).

Note that we took extreme caution to prevent exposure of our colonies to pesticides applied in macadamia plantations. However, foragers might still have been indirectly affected (e.g. when collecting resin from branches previously sprayed). If bees were exposed to pesticides in plantations, this might have amplified the negative impact of low plant diversity in these habitats. In gardens (where pesticides may also have been applied by owners) and at agricultural sites with comparatively high plant species richness, the negative pesticide effect was likely compensated by higher resource diversity and availability, as has been shown for bumblebees^56^.

More biodiverse habitats typically provide more abundant^17,57^ and a broader spectrum of food resources over time^28,32,58^. This is particularly true for human altered habitats (e.g. gardens), where bees can collect food and other resources from both native and exotic flowering plants^59–61^. High plant diversity further offers a wider range of flowering phenologies, thus providing a continuous floral resource supply across seasons which can bridge periods with otherwise low food availability^20,62^.

In contrast to plant species richness, resource abundance rarely affected colony fitness and thus colony population growth (Supplementary Table S4). However, note that resource abundance, as measured in our study, weighed abundant and large plant species (e.g. trees) more heavily than rare or herbaceous plants^32^. Thus, resource abundance was high at sites with many large plant species and thus periodically high resource supply (e.g. agricultural plantations, forests) which may not capture the full picture. More detailed plant data, e.g. on plant coverage^63^ or seasonal availability^20^ would clearly improve deductions on resource abundance.

**Figure 3:**
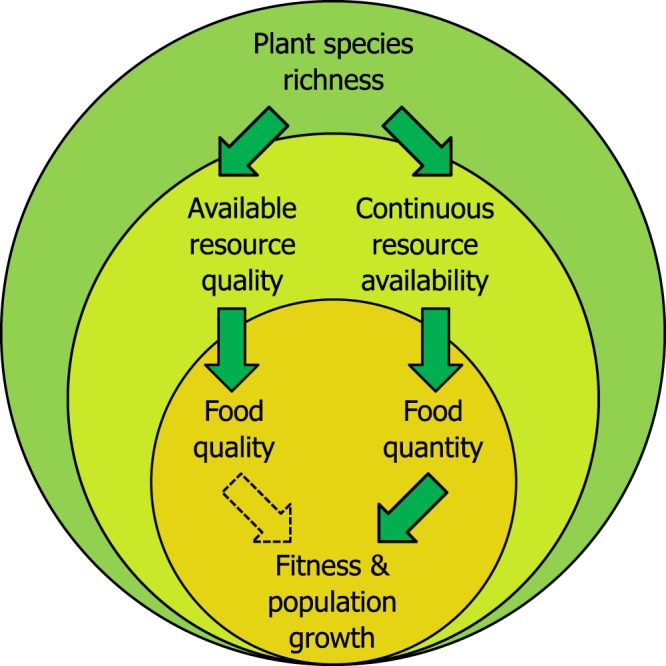
Summary of mechanisms driving social bee fitness and thus colony population growth as revealed by our study. High plant species richness in a habitat (*dark green*) ensures continuous availability and increases overall (nutritional) quality of available resources (*light green*). Continuous resource availability increases foraging activity and thus resource intake by bees resulting in an increased quantity and nutritional quality of stored food (*dark yellow*). Increased quantity of food stores enhances colony fitness and reproduction (i.e. brood volume, queen- and worker production), resulting in larger populations. In biodiverse environments, resource quality does not limit bee fitness.

The strong influence of plant species richness, but not resource abundance, further suggests that a continuous resource supply (as typically provided in biodiverse habitats) may be more important than peaks of high food resource abundance. In fact, *T*. *carbonaria* doubled its food resource intake in highly diverse habitats (e.g. gardens) by maintaining a continuously high foraging activity^31^. This is to say, a continuous resource supply resulted in continuously high food intake, which increased the quantity of stored food resources. This likely allowed colonies to rear and maintain larger colonies, which in turn enhanced foraging activity^31^ and thus foraging success, resulting in an overall improved colony performance largely driven by surrounding plant diversity (Fig. 3). In fact, diverse resources a) enable bees to utilize more food sources at a given time point and over time^20,62^, and b) increase the nutritional quality of accumulated stores through diluting toxic plant compounds or composing a nutritionally balanced diet, as shown by^64–66^. Unexpectedly, food nutritional quality itself did not significantly affect fitness of our colonies (Supplementary Table S4), in contrast to stored food quantity. Pollen protein (i.e. total amino acid content) was even highest in pollen stores from colonies in agricultural habitats, showing that specific plant species (e.g. macadamia) can provide pollen of high protein content, but that available resource diversity does not necessarily correlate with high nutrient contents of collected resources^32^. This may at first seem surprising as previous studies suggested a positive correlation between resource nutrient (e.g. pollen protein) content and bee health^67–69^. However, more recent studies indicate that species-specific target ratios of various micro- and macro-nutrients may be even more important for the health and fitness of animals in general^70^ and bees in particular^71,72^ than overall nutrient contents. Such ideal nutrient ratios may be most easily composed in habitats with high resource diversity where animals can mix resources with variable nutrient contents obtained from different plant species. Moreover, although resource quality generally explained variation in fitness less than resource quantity, we found phosphorus and other micro-nutrient minerals in pollen as well as sucrose in honey to increase with plant species richness (Supplementary Information SM 1 and 2), indicating a generally positive effect of plant diversity on the quality of allocated resources (Fig. 3). Limitations in food quality may thus only become apparent when specific micro- or macro-nutrients are generally limited so that they cannot be compensated by diversified consumption^70^. This may determine colony fitness when resource diversity is reduced, e.g. through increased brood worker mortality due to nutritionally unbalanced cell provisions^72^. Moreover, while we covered several quality measures for pollen and honey, we may have overlooked other important indicators of resource quality (e.g. antioxidants^73^; sterols^74^) or the effect of other non-food resources (e.g. resin:^75^).

Interestingly, we found no effect of plant species richness, food storage quantity or quality on individual bee fitness (Supplementary Table S4). Both worker body size and body fat were highly conserved across different habitats. Note that, unlike honey bees or bumble bees, stingless bees do not progressively feed brood, but mass provision cells once and then seal them^26^. Consequently, *T*. *carbonaria* (and perhaps other social bee species) adjust brood number and thus colony size instead of individual worker fitness to compensate for changes in resource diversity.

Our experiment highlights the importance of biodiverse environments that provide a continuous supply of (floral) resources for the fitness and population growth of bee pollinators. Although our results cannot be readily applied to solitary bee species, in particular floral resource specialists, it is likely that they suffer even more from reduced floral diversity in particular when they lose their only source of forage. Our findings likely also apply to other generalist foragers with relatively long activity periods, e.g. many herbivorous insect species. As these further provide a major food source for several higher trophic levels (e.g. birds), resource diversity induced fitness benefits of lower trophic levels may well translate into increased population sizes of higher trophic levels. Protecting and enhancing biodiversity *per se* can consequently enhance the fitness and population stability of not only social bee species but also entire ecosystem communities. This should subsequently contribute to the stability of the ecosystem by sustaining ecosystem functions facilitated by these organisms, such as plant pollination by bees.

## Electronic supplementary material

Supplementary information
