# Supplementary material for: Social bees are fitter in more biodiverse environments
Source: Sci Rep. 2018 Aug 17;8:12353. doi: 10.1038/s41598-018-30126-0 (PMC6098141; doi:10.1038/s41598-018-30126-0)
Supplement: Supplementary file 1 — Supplementary information [file 41598_2018_30126_MOESM1_ESM.docx]

**Title: Social bees are fitter in more biodiverse environments**

**Short title: Biodiversity benefits bees**

**Authors:** Benjamin F. Kaluza^1,2,7^, Helen M. Wallace^2^, Tim A. Heard^3^, Vanessa Minden^4,5^, Alexandra Klein^6^, Sara D. Leonhardt^7*^

**Affiliations:**

^1^Department of Ecology, Leuphana University, 21335 Lüneburg, Germany, e-mail: *benjamin.kaluza@uni-wuerzburg.de*

^2^Genecology Research Centre, Faculty of Science, Health, Education and Engineering, University of the Sunshine Coast, Maroochydore 4558, Australia, email: *hwallace@usc.edu.au*

^3^CSIRO Ecosystem Sciences, Brisbane 4001, Queensland, Australia, email: *timheard22@gmail.com*

^4^Institute of Biology and Environmental Sciences, University of Oldenburg, 26111 Oldenburg, Germany, email: *vanessa.minden@uni-oldenburg.de*

^5^Department of Biology, Ecology and Evolution, Vrije Universiteit Brussel, 1050 Brussels, Belgium, email: *vanessa.minden@vub.ac.be*

^6^Department of Nature Conservation and Landscape Ecology, University of Freiburg, 79085 Freiburg, Germany, email: *alexandra.klein@nature.uni-freiburg.de*

^7^Department of Animal Ecology and Tropical Biology, University of Würzburg, 97074 Würzburg, Germany, email: *sara.leonhardt@uni-wuerzburg.de*

*Corresponding author: Sara Diana Leonhardt, Department of Animal Ecology and Tropical Biology, University of Würzburg, 97074 Würzburg, Germany, telephone: +49 931 3180168, email: [*sara.leonhardt@uni-wuerzburg.de*](mailto:sara.leonhardt@uni-wuerzburg.de)

**Supplementary Material**

**SM 1. Correlation matrix**

Spearman rank corellation matrix of explanatory variables related to plant diversity and food quantity and nutritional quality and of fitness response variables (Table S4). Abbreviations as follows: ***pRi***: plant species richness; ***pAb***: plant resource abundance; ***G_A_***: garden area; ***F_A_***: forest area; ***P_A_***: plantation area; ***rQt***: resource quantity (weight pollen and honey stores); ***Pr_P_***: total protein in pollen; ***eAA_P_***: essential amino acids in pollen; ***AA_P_***: amino acid content (first PCA axis); ***P_P_***: phosphorus in pollen; ***N_P_***: nitrogen in pollen; ***C_P_***: carbon in pollen; ***mn_P_***: micro elements in pollen (first PCA axis); ***S_H_***: honey sucrose content; ***Wa_H_***: honey water content; ***pH***: honey acidity; ***V_B_***: brood volume; ***C_B_***: brood circumference; ***Q_P_***: number of queen pupae; ***W_C_***: number of open worker cells; ***W_F_***: worker body fat.

**Table S1a).** Spearman correlations with plant diversity-related variables. Asterisks indicate significant correlations: * *P* < 0.05, ** *P* < 0.01, *** *P* < 0.001.

|  | ***pRi*** | | ***pAb*** | | ***G_A_*** | | ***F_A_*** | | ***P_A_*** | |
| --- | --- | --- | --- | --- | --- | --- | --- | --- | --- | --- |
| ***pAb*** | -0.11 |  |  |  |  |  |  |  |  |  |
| ***G_A_*** | 0.76 | *** | -0.29 | ** |  |  |  |  |  |  |
| ***F_A_*** | -0.38 | *** | 0.88 | *** | -0.63 | *** |  |  |  |  |
| ***P_A_*** | -0.69 | *** | -0.13 |  | -0.39 | *** | -0.06 |  |  |  |
| ***rQt*** | 0.50 | *** | -0.22 | * | 0.52 | *** | -0.35 | *** | -0.26 | ** |
| ***Pr_P_*** | -0.09 |  | -0.16 |  | 0.08 |  | -0.19 |  | 0.27 | ** |
| ***eAA_P_*** | -0.09 |  | -0.18 |  | 0.06 |  | -0.20 |  | 0.29 | ** |
| ***AA_P_*** | -0.14 |  | -0.17 |  | 0.02 |  | -0.17 |  | 0.32 | ** |
| ***P_P_*** | 0.33 | ** | -0.33 | ** | 0.44 | *** | -0.46 | *** | 0.03 |  |
| ***N_P_*** | 0.12 |  | 0.10 |  | 0.11 |  | 0.01 |  | 0.02 |  |
| ***C_P_*** | -0.02 |  | 0.06 |  | -0.04 |  | 0.01 |  | 0.15 |  |
| ***mn_P_*** | 0.48 | *** | -0.17 |  | 0.47 | *** | -0.30 | ** | -0.32 | ** |
| ***S_H_*** | 0.21 | * | -0.37 | *** | 0.40 | *** | -0.46 | *** | -0.07 |  |
| ***Wa_H_*** | -0.24 | * | 0.42 | *** | -0.36 | *** | 0.47 | *** | 0.13 |  |
| ***pH*** | 0.02 |  | -0.16 |  | 0.04 |  | -0.06 |  | -0.23 | * |
| ***V_B_*** | 0.54 | *** | -0.21 |  | 0.44 | *** | -0.32 | ** | -0.30 | ** |
| ***C_B_*** | 0.48 | *** | -0.11 |  | 0.33 | *** | -0.20 | * | -0.23 | * |
| ***Q_P_*** | 0.30 | ** | -0.13 |  | 0.23 | * | -0.15 |  | -0.23 | * |
| ***W_C_*** | 0.37 | ** | -0.09 |  | 0.19 |  | -0.18 |  | -0.08 |  |
| ***W_F_*** | 0.17 |  | -0.07 |  | 0.01 |  | -0.04 |  | -0.16 |  |

**Table S1b)**. Spearman correlations with food quantity and nutritional quality variables (aberrations as above).

|  | ***W_PH_*** | | ***Pr_P_*** | | ***eAA_P_*** | | ***AA_P_*** | | ***P_P_*** | | ***N_P_*** | | ***C_P_*** | | ***mn_P_*** | | ***S_H_*** | | ***Wa_H_*** | | ***pH*** | |
| --- | --- | --- | --- | --- | --- | --- | --- | --- | --- | --- | --- | --- | --- | --- | --- | --- | --- | --- | --- | --- | --- | --- |
| ***Pr_P_*** | 0.07 |  |  |  |  |  |  |  |  |  |  |  |  |  |  |  |  |  |  |  |  |  |
| ***eAA_P_*** | 0.06 |  | 0.98 | *** |  |  |  |  |  |  |  |  |  |  |  |  |  |  |  |  |  |  |
| ***AA_P_*** | 0.03 |  | 0.98 | *** | 0.99 | *** |  |  |  |  |  |  |  |  |  |  |  |  |  |  |  |  |
| ***P_P_*** | 0.20 |  | 0.40 | *** | 0.37 | *** | 0.35 | *** |  |  |  |  |  |  |  |  |  |  |  |  |  |  |
| ***N_P_*** | -0.02 |  | 0.52 | *** | 0.52 | *** | 0.50 | *** | 0.45 | *** |  |  |  |  |  |  |  |  |  |  |  |  |
| ***C_P_*** | -0.07 |  | 0.17 |  | 0.22 | * | 0.20 |  | 0.04 |  | 0.53 | *** |  |  |  |  |  |  |  |  |  |  |
| ***mn_P_*** | 0.29 | ** | -0.17 |  | -0.19 |  | -0.19 |  | 0.25 | * | -0.06 |  | -0.19 |  |  |  |  |  |  |  |  |  |
| ***S_H_*** | 0.28 | ** | -0.02 |  | -0.01 |  | -0.03 |  | 0.21 | * | -0.16 |  | -0.06 |  | 0.15 |  |  |  |  |  |  |  |
| ***Wa_H_*** | -0.26 | * | 0.11 |  | 0.11 |  | 0.12 |  | -0.21 | * | 0.20 |  | 0.14 |  | -0.18 |  | -0.95 |  |  |  |  |  |
| ***pH*** | 0.06 |  | -0.10 |  | -0.13 |  | -0.12 |  | -0.01 |  | -0.15 |  | -0.16 |  | -0.05 |  | 0.57 | *** | -0.61 | *** |  |  |
| ***V_B_*** | 0.63 | *** | 0.06 |  | 0.05 |  | 0.02 |  | 0.16 |  | 0.11 |  | 0.06 |  | 0.35 | ** | 0.30 | ** | -0.28 | * | 0.02 |  |
| ***C_B_*** | 0.56 | *** | 0.07 |  | 0.06 |  | 0.04 |  | 0.15 |  | 0.13 |  | 0.11 |  | 0.15 |  | 0.29 | ** | -0.27 | ** | 0.12 |  |
| ***Q_P_*** | 0.49 | *** | -0.02 |  | -0.03 |  | -0.06 |  | 0.02 |  | -0.03 |  | 0.18 |  | 0.10 |  | 0.18 |  | -0.15 |  | 0.08 |  |
| ***W_C_*** | 0.19 |  | -0.07 |  | -0.06 |  | -0.06 |  | 0.11 |  | 0.09 |  | 0.10 |  | 0.19 |  | 0.06 |  | -0.06 |  | 0.03 |  |
| ***W_F_*** | -0.15 |  | 0.16 |  | 0.21 |  | 0.19 |  | 0.00 |  | 0.32 | ** | 0.40 | *** | -0.03 |  | 0.03 |  | 0.03 |  | -0.11 |  |

**Table S1c)**. Spearman correlations between fitness response variables (aberrations as above).

|  | ***V_B_*** | | ***C_B_*** | | ***Q_P_*** | | ***W_C_*** | |
| --- | --- | --- | --- | --- | --- | --- | --- | --- |
| ***C_B_*** | 0.85 | *** |  |  |  |  |  |  |
| ***Q_P_*** | 0.49 | *** | 0.57 | *** |  |  |  |  |
| ***W_C_*** | 0.17 |  | 0.40 | ** | 0.32 | ** |  |  |
| ***W_F_*** | 0.02 |  | -0.02 |  | -0.01 |  | 0.05 |  |

**SM 2. Food quantity and nutritional quality**

*Tetragonula carbonaria* stores honey and pollen in separate pots [^1^](#_ENREF_1). We collected honey and pollen samples from 1-10 pots of varying age from each colony to determine food nutritional quality. Honey samples were analyzed for their sucrose and water content using hand-held refractometers (sucrose: Eclipse Refractometer, Bellingham + Stanley Ltd., Lawrenceville, USA; water: HHR-2N Honey Refractometer, ATAGO Co. Ltd., Tokyo, Japan). Honey acidity was further measured using standard pH-test strips.

Amino acid contents in pollen samples were analyzed by ion exchange chromatography (IEC: Biotronik, amino acid analyzer LC 3000) as described in Kaluza*, et al.* [^2^](#_ENREF_2) and Leonhardt and Blüthgen [^3^](#_ENREF_3). Total protein was measured as total amino acid concentration (mg/g) and obtained by summing the molar masses of respective amino acids. Methionine, arginine, lysine, isoleucine, leucine, phenylalanine, histidine, valine and threonine and proline [^4^](#_ENREF_4)^,^[^5^](#_ENREF_5) were considered essential amino acids for bees. A PCA was performed on all single amino acids and the first axis (explaining 80% of the variance across samples) used as parameter for pollen amino acid content in further statistical analysis (Table S2). We additionally used total protein (mg/g) and the sum of all essential amino acids (mg/g) as response variables in statistical analyses (Table S2), because bees appear to be primarily affected by overall protein content rather than amino acid composition of pollen [reviewed by ^6^](#_ENREF_6).

We further performed a stoichiometric analysis on pollen following Minden and Kleyer [^7^](#_ENREF_7). All pollen material was milled at 300–400 revolutions (‘pulverisette 7’; Fritsch, Idar-Oberstein, Germany) and dried at 70°C for 4–5h. To analyze carbon and nitrogen (C and N) 2–3 mg of material were placed in tin tubes (0.1 mg precision balance CP 225 D; Sartorius, Göttingen, Germany) and analyzed using a CHNS Analyser Flash EA (Thermo Electron Corp., Waltham, MA, USA). All other elements (K, S, Mg, Ca, Na, P, Fe, B, Cu) were analyzed using optical emission spectrometry with inductively coupled plasma (iCAP 6000, Thermo Scientific), for which 8-10 mg material were processed with nitric acid and hydrogen peroxide and subsequently measured [^8^](#_ENREF_8).

Stoichiometric analysis of elements were performed for nest pollen of each study site and boron, calcium, copper, iron, potassium, magnesium, manganese, sodium, sulfur and zinc were considered micro-elements (Table S3). A principal component analysis was performed on all micro-elements, and the first axis of the PCA (explaining 87% of the variance across samples) was used for further analyses. The macro-elements phosphorus, nitrogen and carbon were entered as separate factors in the correlation analyses (see SM 1).

**Table S2.** Amino acids measured in nest pollen (mean ± sd [mg/g]) across all colonies at a study site (forests: F1a-F4b; gardens: G1a-G4b and plantations: P1a-P4b). Amino acids in bold are considered essential (pooled under total essential amino acids (AAs)). Dashes indicate that an amino acid could not be detected in this sample.

| **Amino Acid** | **F1a** | **F1b** | **F2a** | **F2b** | **F3a** | **F3b** | **F4a** | **F4b** |
| --- | --- | --- | --- | --- | --- | --- | --- | --- |
| Asparagine acid | 12.85 ± 3.56 | 11.97 ± 6.99 | 10.45 ± 2.28 | 10.19 ± 2.03 | 11.44 ± 2.66 | 11.25 ± 1.66 | 12.17 ± 1.78 | 12.52 ± 3.14 |
| Hydroxyproline | 1.09 ± 1.43 | 1.88 ± 0.77 | 0.43 ± 0.75 | 2.29 ± 2.65 | 0.77 ± 0.60 | 0.21 ± 0.42 | 0.58 ± 0.72 | 1.03 ± 0.86 |
| **Threonine** | 4.81 ± 1.87 | 4.16 ± 2.42 | 3.57 ± 0.37 | 3.86 ± 0.49 | 4.07 ± 1.08 | 4.23 ± 0.56 | 4.99 ± 0.83 | 5.36 ± 1.53 |
| Serine | 7.40 ± 2.12 | 6.25 ± 3.27 | 5.57 ± 0.85 | 6.22 ± 0.47 | 6.20 ± 1.42 | 5.87 ± 0.53 | 7.33 ± 0.90 | 7.43 ± 2.09 |
| Glutamic acid | 10.82 ± 3.24 | 9.54 ± 5.77 | 8.05 ± 1.69 | 8.37 ± 1.30 | 9.10 ± 3.04 | 8.04 ± 1.79 | 9.95 ± 2.32 | 10.16 ± 3.64 |
| a-Aminoadipic acid | 0 | 0 | 0 | 0 | 0 | 0 | 0.66 ± 0.85 | 0 |
| **Proline** | 16.01 ± 5.05 | 9.83 ± 4.62 | 14.17 ± 2.25 | 11.47 ± 3.71 | 10.85 ± 1.38 | 11.00 ± 3.13 | 15.24 ± 3.58 | 16.73 ± 6.05 |
| Glycine | 7.14 ± 1.71 | 5.49 ± 2.90 | 5.31 ± 0.59 | 5.29 ± 0.79 | 5.91 ± 1.31 | 5.61 ± 0.65 | 6.50 ± 0.92 | 6.64 ± 1.94 |
| Alanine | 7.98 ± 2.13 | 6.43 ± 3.30 | 5.93 ± 0.87 | 6.04 ± 0.68 | 6.83 ± 1.40 | 6.85 ± 0.81 | 7.28 ± 0.87 | 7.52 ± 1.90 |
| **Valine** | 3.44 ± 1.29 | 2.81 ± 1.64 | 2.47 ± 0.33 | 2.47 ± 0.31 | 2.72 ± 0.60 | 2.93 ± 0.36 | 3.11 ± 0.45 | 3.37 ± 0.79 |
| Cysteine | 0.45 ± 0.56 | 0.50 ± 0.57 | 0 | 0.10 ± 0.20 | 0.46 ± 0.31 | 0 | 0.46 ± 0.37 | 0.58 ± 0.14 |
| **Methionine** | 1.98 ± 0.73 | 1.80 ± 1.07 | 1.49 ± 0.12 | 1.3 ± 0.42 | 1.75 ± 0.52 | 1.74 ± 0.40 | 2.03 ± 0.32 | 2.15 ± 0.73 |
| **Isoleucine** | 2.61 ± 0.85 | 2.20 ± 1.13 | 2.04 ± 0.48 | 2.04 ± 0.39 | 1.96 ± 0.29 | 2.19 ± 0.20 | 2.46 ± 0.34 | 2.58 ± 0.63 |
| **Leucine** | 8.54 ± 2.37 | 7.14 ± 3.58 | 6.41 ± 1.29 | 6.69 ± 1.40 | 6.92 ± 0.84 | 7.06 ± 0.86 | 8.05 ± 1.27 | 8.20 ± 2.10 |
| Tyrosine | 2.78 ± 0.98 | 2.13 ± 1.45 | 1.73 ± 0.39 | 1.94 ± 0.40 | 2.22 ± 0.62 | 2.54 ± 0.38 | 2.56 ± 0.44 | 2.82 ± 0.70 |
| beta-Alanine | 0 | 0.19 ± 0.37 | 0 | 0 | 0.16 ± 0.33 | 0 | 0 | 0 |
| **Phenylalanine** | 4.42 ± 1.33 | 3.70 ± 2.05 | 3.43 ± 0.81 | 3.35 ± 0.69 | 3.55 ± 0.64 | 3.54 ± 0.52 | 4.16 ± 0.63 | 4.34 ± 1.26 |
| Gamma amino butyric acid | 0.92 ± 0.19 | 0.62 ± 0.18 | 0.72 ± 0.29 | 0.60 ± 0.20 | 0.74 ± 0.05 | 0.79 ± 0.31 | 0.80 ± 0.22 | 0.85 ± 0.22 |
| Ornithine | 0 | 0 | 0 | 0 | 0 | 0 | 0 | 0 |
| **Lysine** | 7.48 ± 1.96 | 7.08 ± 3.91 | 6.05 ± 2.21 | 6.79 ± 1.03 | 7.18 ± 1.23 | 7.01 ± 0.84 | 8.92 ± 1.43 | 8.17 ± 2.49 |
| **Histidine** | 3.02 ± 1.12 | 2.59 ± 1.35 | 1.99 ± 0.19 | 2.62 ± 0.59 | 2.24 ± 0.54 | 2.20 ± 0.17 | 2.59 ± 0.38 | 2.93 ± 0.81 |
| **Arginine** | 7.04 ± 2.90 | 5.43 ± 4.05 | 4.32 ± 1.21 | 3.91 ± 0.99 | 5.21 ± 1.50 | 5.29 ± 0.80 | 4.87 ± 0.69 | 5.50 ± 1.22 |
|  |  |  |  |  |  |  |  |  |
| Total protein | 110.77 ± 33.10 | 91.75 ± 49.29 | 84.15 ± 16.10 | 85.53 ± 11.36 | 90.28 ± 18.40 | 88.35 ± 11.45 | 104.71 ± 14.78 | 108.85 ± 30.14 |
| **Total essential AAs** | 59.35 ± 18.56 | 46.75 ± 25.27 | 45.95 ± 8.85 | 44.49 ± 8.33 | 46.44 ± 8.34 | 47.19 ± 6.40 | 56.42 ± 8.94 | 59.31 ± 17.17 |

**Table S2** continued.

| **Amino Acid** | **G1a** | **G1b** | **G2a** | **G2b** | **G3a** | **G3b** | **G4a** | **G4b** |
| --- | --- | --- | --- | --- | --- | --- | --- | --- |
| Asparagine acid | 14.94 ± 6.77 | 14.35 ± 0.06 | 11.18 ± 1.9 | 12.83 ± 2.14 | 14.55 ± 4.04 | 15.92 ± 3.20 | 11.95 ± 3.48 | 11.25 ± 1.38 |
| Hydroxyproline | 0.54 ± 0.77 | 0.55 ± 0.62 | 0 | 0.54 ± 0.54 | 0.14 ± 0.32 | 0.52 ± 0.74 | 0.33 ± 0.45 | 0.31 ± 0.37 |
| **Threonine** | 6.17 ± 3.38 | 5.24 ± 0.58 | 3.93 ± 0.53 | 5.17 ± 1.07 | 5.10 ± 1.53 | 5.97 ± 1.21 | 4.94 ± 1.68 | 4.17 ± 0.70 |
| Serine | 8.32 ± 3.84 | 7.25 ± 0.74 | 5.92 ± 1.12 | 6.85 ± 1.03 | 7.21 ± 1.74 | 8.45 ± 1.48 | 6.67 ± 2.07 | 6.11 ± 0.81 |
| Glutamic acid | 11.84 ± 4.56 | 11.05 ± 0.55 | 9.37 ± 2.17 | 10.57 ± 1.69 | 11.61 ± 3.61 | 5.93 ± 6.44 | 9.98 ± 3.32 | 9.17 ± 1.61 |
| a-Aminoadipic acid | 0.15 ± 0.21 | 0 | 0 | 0 | 0 | 0 | 0 | 0 |
| **Proline** | 13.37 ± 7.50 | 13.37 ± 3.97 | 8.05 ± 1.76 | 10.40 ± 4.00 | 14.57 ± 4.33 | 14.87 ± 0.90 | 12.31 ± 1.88 | 0 |
| Glycine | 7.22 ± 2.45 | 6.40 ± 0.65 | 5.82 ± 1.17 | 6.22 ± 0.94 | 6.56 ± 1.46 | 7.42 ± 1.56 | 6.21 ± 1.79 | 5.81 ± 0.81 |
| Alanine | 9.04 ± 3.40 | 7.95 ± 0.90 | 7.11 ± 1.45 | 7.53 ± 1.09 | 8.09 ± 1.87 | 9.43 ± 1.91 | 7.39 ± 2.23 | 6.89 ± 0.98 |
| **Valine** | 4.29 ± 2.21 | 3.56 ± 0.33 | 2.85 ± 0.39 | 3.34 ± 0.63 | 3.49 ± 0.96 | 3.97 ± 0.73 | 3.33 ± 1.06 | 2.90 ± 0.53 |
| Cysteine | 0.79 ± 1.12 | 0.49 ± 0.07 | 0.22 ± 0.20 | 0.47 ± 0.46 | 0.43 ± 0.26 | 0.42 ± 0.60 | 0.21 ± 0.42 | 0.38 ± 0.34 |
| **Methionine** | 2.76 ± 1.51 | 2.26 ± 0.34 | 2.09 ± 0.60 | 2.01 ± 0.34 | 2.22 ± 0.62 | 2.59 ± 0.42 | 2.00 ± 0.62 | 1.93 ± 0.33 |
| **Isoleucine** | 3.65 ± 1.89 | 2.68 ± 0.37 | 2.24 ± 0.37 | 2.63 ± 0.36 | 2.76 ± 0.90 | 3.17 ± 0.48 | 2.66 ± 0.93 | 2.26 ± 0.38 |
| **Leucine** | 10.36 ± 3.83 | 8.47 ± 1.01 | 7.52 ± 1.45 | 8.36 ± 1.15 | 8.94 ± 2.69 | 9.65 ± 0.98 | 8.03 ± 2.48 | 7.37 ± 1.26 |
| Tyrosine | 3.71 ± 1.87 | 2.92 ± 0.36 | 2.27 ± 0.55 | 2.86 ± 0.65 | 2.95 ± 0.90 | 3.10 ± 0.21 | 2.83 ± 1.07 | 2.45 ± 0.49 |
| beta-Alanine | 0 | 0 | 0 | 0 | 0 | 0.37 ± 0.52 | 0 | 0 |
| **Phenylalanine** | 5.59 ± 2.08 | 4.51 ± 0.56 | 3.86 ± 0.84 | 4.42 ± 0.62 | 4.67 ± 1.48 | 5.13 ± 0.59 | 4.30 ± 1.47 | 3.75 ± 0.66 |
| Gamma amino butyric acid | 0.73 ± 0.41 | 0.99 ± 0.13 | 0.46 ± 0.02 | 0.58 ± 0.25 | 0.94 ± 0.31 | 1.04 ± 0.20 | 0.70 ± 0.15 | 0.79 ± 0.29 |
| Ornithine | 0 | 0 | 0 | 0 | 0 | 0 | 0 | 0 |
| **Lysine** | 9.95 ± 4.04 | 8.06 ± 1.30 | 7.33 ± 1.76 | 7.73 ± 1.55 | 9.03 ± 3.18 | 9.93 ± 1.68 | 7.50 ± 2.53 | 7.08 ± 1.05 |
| **Histidine** | 3.47 ± 1.99 | 2.75 ± 0.20 | 2.39 ± 0.47 | 2.70 ± 0.51 | 2.94 ± 0.57 | 3.59 ± 0.01 | 2.69 ± 0.86 | 2.65 ± 0.29 |
| **Arginine** | 7.86 ± 4.43 | 6.10 ± 0.48 | 5.45 ± 1.18 | 6.18 ± 1.14 | 6.28 ± 1.67 | 6.52 ± 1.02 | 5.48 ± 1.92 | 5.13 ± 0.94 |
|  |  |  |  |  |  |  |  |  |
| Total protein | 124.77 ± 58.26 | 108.96 ± 3.87 | 88.09 ± 15.05 | 101.38 ± 17.11 | 112.49 ± 31.36 | 117.97 ± 9.81 | 99.51 ± 29.43 | 92.64 ± 12.24 |
| **Total essential AAs** | 67.48 ± 32.86 | 57.01 ± 1.14 | 45.73 ± 7.1 | 52.93 ± 9.46 | 60.01 ± 17.7 | 65.38 ± 6.23 | 53.24 ± 15.13 | 49.49 ± 6.81 |

**Table S2** continued.

| **Amino Acid** | **P1a** | **P2a** | **P2b** | **P3a** | **P3b** | **P4a** | **P4b** |
| --- | --- | --- | --- | --- | --- | --- | --- |
| Asparagine acid | 13.42 ± 3.09 | 12.35 ± 1.05 | 12.00 ± 5.54 | 13.94 ± 3.51 | 12.83 ± 3.91 | 14.4 ± 4.93 | 11.32 ± 1.81 |
| Hydroxyproline | 0.71 ± 1.05 | 0.16 ± 0.27 | 0.24 ± 0.41 | 0.13 ± 0.29 | 0.16 ± 0.32 | 0.66 ± 0.98 | 1.14 ± 0.89 |
| **Threonine** | 5.06 ± 1.68 | 4.45 ± 0.74 | 4.66 ± 2.57 | 5.40 ± 1.26 | 4.97 ± 1.74 | 5.71 ± 2.33 | 4.35 ± 0.55 |
| Serine | 7.49 ± 1.83 | 7.03 ± 0.87 | 6.94 ± 3.40 | 8.10 ± 1.75 | 7.54 ± 2.37 | 8.52 ± 3.25 | 6.82 ± 1.04 |
| Glutamic acid | 12.78 ± 3.45 | 11.83 ± 2.21 | 12.08 ± 5.24 | 13.63 ± 3.19 | 12.74 ± 3.79 | 13.1 ± 6.31 | 9.98 ± 1.75 |
| a-Aminoadipic acid | 0 | 0 | 0 | 0 | 0 | 0.36 ± 0.44 | 0.88 ± 0.50 |
| **Proline** | 17.98 ± 4.49 | 19.67 ± 3.23 | 18.02 ± 12.71 | 18.71 ± 6.67 | 17.34 ± 6.17 | 17.54 ± 11.5 | 15.15 ± 3.27 |
| Glycine | 7.24 ± 1.38 | 6.87 ± 0.93 | 6.78 ± 3.25 | 7.92 ± 1.87 | 7.28 ± 2.26 | 7.75 ± 2.94 | 6.23 ± 0.86 |
| Alanine | 7.98 ± 1.47 | 7.40 ± 0.66 | 7.61 ± 2.67 | 8.66 ± 1.98 | 7.94 ± 2.47 | 8.53 ± 3.11 | 6.99 ± 1.17 |
| **Valine** | 3.54 ± 0.97 | 3.17 ± 0.33 | 3.17 ± 1.36 | 3.72 ± 0.84 | 3.47 ± 1.13 | 3.75 ± 1.40 | 2.94 ± 0.45 |
| Cysteine | 0.33 ± 0.39 | 0.21 ± 0.36 | 0.11 ± 0.18 | 0.40 ± 0.42 | 0.45 ± 0.34 | 0.48 ± 0.35 | 0.36 ± 0.25 |
| **Methionine** | 2.46 ± 0.64 | 2.35 ± 0.33 | 2.29 ± 1.38 | 2.84 ± 0.71 | 2.52 ± 0.94 | 2.79 ± 1.17 | 2.05 ± 0.32 |
| **Isoleucine** | 2.76 ± 0.65 | 2.48 ± 0.27 | 2.53 ± 1.21 | 2.84 ± 0.54 | 2.64 ± 0.95 | 3.00 ± 1.14 | 2.32 ± 0.49 |
| **Leucine** | 8.88 ± 1.90 | 8.17 ± 0.69 | 7.90 ± 3.76 | 9.40 ± 1.94 | 8.99 ± 3.19 | 9.12 ± 3.55 | 7.60 ± 1.64 |
| Tyrosine | 2.47 ± 0.70 | 2.23 ± 0.06 | 2.20 ± 1.18 | 2.79 ± 0.50 | 2.88 ± 1.04 | 2.91 ± 1.16 | 2.14 ± 0.37 |
| beta-Alanine | 0 | 0 | 0 | 0.26 ± 0.59 | 0 | 0 | 0 |
| **Phenylalanine** | 4.59 ± 1.02 | 4.37 ± 0.43 | 4.34 ± 2.25 | 5.02 ± 1.12 | 4.64 ± 1.65 | 4.93 ± 2.06 | 3.97 ± 0.83 |
| Gamma amino butyric acid | 0.73 ± 0.41 | 0.86 ± 0.34 | 0.95 ± 0.33 | 0.7 ± 0.46 | 0.73 ± 0.51 | 0.61 ± 0.27 | 0.89 ± 0.51 |
| Ornithine | 0 | 0 | 0 | 0 | 0.07 ± 0.14 | 0 | 0 |
| **Lysine** | 8.05 ± 1.02 | 7.48 ± 1.54 | 8.18 ± 4.24 | 9.29 ± 2.21 | 8.28 ± 2.82 | 10.19 ± 4.12 | 8.57 ± 1.86 |
| **Histidine** | 3.30 ± 0.48 | 2.82 ± 0.44 | 3.15 ± 1.30 | 3.52 ± 0.77 | 3.20 ± 1.15 | 3.54 ± 1.35 | 2.89 ± 0.63 |
| **Arginine** | 5.13 ± 1.19 | 4.81 ± 0.32 | 4.60 ± 2.60 | 6.6 ± 2.64 | 6.90 ± 3.93 | 6.52 ± 2.77 | 4.46 ± 0.93 |
|  |  |  |  |  |  |  |  |
| Total protein | 114.98 ± 23.79 | 108.7 ± 13.44 | 107.75 ± 53.89 | 123.88 ± 30.83 | 115.58 ± 38.37 | 124.41 ± 50.84 | 101.05 ± 17.13 |
| **Total essential AAs** | 61.76 ± 12.55 | 59.76 ± 7.77 | 58.84 ± 33.18 | 67.35 ± 17.11 | 62.96 ± 22.63 | 67.09 ± 29.99 | 54.31 ± 9.94 |

**Table S3**. Stoichiometric analysis of elements in nest pollen (mean ± sd [mg/g]) per study site (forests: F1a-F4b; gardens: G1a-G4b and plantations: P1a-P4b). Macro-elements (bold) comprise phosphorus, nitrogen and carbon, while micro-elements comprise all other elements. Dashes indicate that an amino acid could not be detected in this sample.

| **Minerals** | **F1a** | **F1b** | **F2a** | **F2b** | **F3a** | **F3b** | **F4a** | **F4b** |
| --- | --- | --- | --- | --- | --- | --- | --- | --- |
| Boron | 0.02 ± 0.02 | 0.03 ± 0.01 | 0.02 | 0.03 ± 0.02 | 0.02 ± 0.01 | 0.01 | 0.04 ± 0.01 | 0.01 ± 0.01 |
| Calcium | 1.40 ± 0.42 | 1.04 ± 0.38 | 1.30 ± 0.81 | 1.44 ± 0.30 | 2.46 ± 1.45 | 2.28 ± 0.28 | 1.63 ± 0.67 | 1.31 ± 0.48 |
| Copper | 0.02 | 0.01 | 0.01 | 0.01 | 0.02 ± 0.01 | 0.02 ± 0.01 | 0.01 | 0.01 |
| Iron | 0.06 ± 0.02 | 0.07 ± 0.02 | 0.04 ± 0.01 | 0.05 ± 0.02 | 0.06 ± 0.01 | 0.05 ± 0.01 | 0.04 ± 0.01 | 0.05 ± 0.01 |
| Potassium | 5.10 ± 0.75 | 4.61 ± 1.03 | 6.06 ± 1.04 | 5.53 ± 0.97 | 6.11 ± 0.84 | 6.41 ± 0.47 | 6.35 ± 0.67 | 6.53 ± 0.81 |
| Magnesium | 0.73 ± 0.12 | 0.70 ± 0.21 | 1.02 ± 0.18 | 0.78 ± 0.26 | 0.96 ± 0.08 | 1.02 ± 0.10 | 1.12 ± 0.22 | 1.07 ± 0.17 |
| Manganese | 0.03 ± 0.01 | 0.02 | 0.06 | 0.05 ± 0.01 | 0.12 ± 0.07 | 0.17 ± 0.11 | 0.05 ± 0.01 | 0.07 ± 0.03 |
| Sodium | 0.44 ± 0.10 | 0.51 ± 0.09 | 0.57 ± 0.09 | 0.48 ± 0.14 | 0.29 ± 0.13 | 0.26 ± 0.13 | 0.21 ± 0.10 | 0.21 ± 0.02 |
| Sulphur | 1.94 ± 0.39 | 1.57 ± 0.84 | 1.49 ± 0.21 | 1.57 ± 0.37 | 2.07 ± 0.30 | 2.00 ± 0.42 | 2.33 ± 0.51 | 1.95 ± 0.38 |
| Zinc |  |  |  |  |  |  |  |  |
| **Phosphorus** | 3.64 ± 0.62 | 3.52 ± 1.82 | 4.14 ± 0.91 | 3.84 ± 0.74 | 4.89 ± 0.53 | 5.24 ± 0.15 | 5.00 ± 0.69 | 5.66 ± 2.17 |
| **Nitrogen** | 34.91 ± 5.69 | 33.17 ± 13.95 | 32.23 ± 0.70 | 27.06 ± 7.29 | 37.75 ± 8.65 | 38.63 ± 7.67 | 40.17 ± 6.27 | 34.72 ± 4.51 |
| **Carbon** | 465.16 ± 34.06 | 488.23 ± 17.91 | 481.23 ± 14.42 | 472.95 ± 112 | 473.64 ± 41.39 | 461.56 ± 39.27 | 524.60 ± 69.27 | 475.98 ± 39.87 |
|  |  |  |  |  |  |  |  |  |
| Micro-elements | 9.60 ± 1.25 | 8.35 ± 1.80 | 10.33 ± 1.18 | 9.76 ± 1.47 | 11.92 ± 2.26 | 11.89 ± 0.65 | 11.57 ± 0.73 | 10.97 ± 1.22 |
| **Macro-elements** | 503.71 ± 36.92 | 524.91 ± 4.56 | 517.60 ± 12.90 | 503.84 ± 118.72 | 516.27 ± 47.81 | 505.44 ± 46.85 | 569.77 ± 74.91 | 516.36 ± 44.65 |

**Table S3** continued.

| **Minerals** | **G1a** | **G1b** | **G2a** | **G2b** | **G3a** | **G3b** | **G4b** | **G4a** |
| --- | --- | --- | --- | --- | --- | --- | --- | --- |
| Boron | 0.02 ± 0.01 | 0.02 | 0.01 | 0.02 ± 0.01 | 0.02 | 0.01 | 0.02 | 0.01 |
| Calcium | 2.26 ± 0.14 | 2.12 ± 0.30 | 2.23 ± 0.59 | 2.13 ± 0.76 | 2.33 ± 0.77 | 1.93 ± 0.17 | 2.28 ± 0.71 | 2.78 ± 1.32 |
| Copper | 0.01 | 0.01 | 0.01 | 0.01 ± 0 | 0.02 ± 0.01 | 0.01 | 0.02 | 0.02 ± 0.01 |
| Iron | 0.08 ± 0.06 | 0.06 ± 0.01 | 0.05 ± 0.01 | 0.05 ± 0.01 | 0.05 ± 0.01 | 0.06 | 0.06 ± 0.02 | 0.05 ± 0.01 |
| Potassium | 6.14 ± 0.54 | 6.73 ± 0.96 | 6.31 ± 0.24 | 6.93 ± 0.60 | 7.50 ± 0.59 | 7.39 ± 0.98 | 7.24 ± 0.44 | 6.65 ± 0.25 |
| Magnesium | 1.44 ± 0.13 | 1.59 ± 0.35 | 1.38 ± 0.02 | 1.38 ± 0.30 | 1.41 ± 0.13 | 1.33 ± 0.10 | 1.50 ± 0.28 | 1.43 ± 0.09 |
| Manganese | 0.04 | 0.04 ± 0.01 | 0.04 ± 0.02 | 0.05 ± 0.02 | 0.03 ± 0.01 | 0.03 | 0.04 ± 0.01 | 0.05 ± 0.01 |
| Sodium | 0.58 ± 0.04 | 0.34 ± 0.12 | 0.40 ± 0.05 | 0.43 ± 0.16 | 0.22 ± 0.07 | 0.19 ± 0.01 | 0.32 ± 0.06 | 0.22 ± 0.06 |
| Sulphur | 2.30 ± 0.09 | 1.71 ± 0.48 | 1.71 ± 0.31 | 1.97 ± 0.30 | 1.90 ± 0.24 | 1.87 ± 0.12 | 2.11 ± 0.29 | 1.96 ± 0.41 |
| Zinc | 0.11 ± 0.14 |  |  |  |  |  |  |  |
| **Phosphorus** | 5.49 ± 0.96 | 5.61 ± 1.23 | 5.51 ± 0.13 | 5.63 ± 0.96 | 5.51 ± 0.36 | 5.56 ± 0.14 | 5.64 ± 1.06 | 5.51 ± 0.25 |
| **Nitrogen** | 36.38 ± 3.78 | 35.13 ± 4.40 | 32.83 ± 1.83 | 33.69 ± 2.76 | 36.69 ± 1.50 | 40.19 ± 0.66 | 41.97 ± 21.24 | 33.72 ± 5.89 |
| **Carbon** | 486.45 ± 5.56 | 447.55 ± 40.66 | 500.87 ± 15.17 | 467.32 ± 23.93 | 485.53 ± 16.16 | 490.46 ± 1.54 | 480.38 ± 21.21 | 465.42 ± 50.19 |
|  |  |  |  |  |  |  |  |  |
| Micro-elements | 12.97 ± 0.80 | 12.43 ± 2.26 | 11.85 ± 0.42 | 12.67 ± 1.37 | 13.24 ± 1.52 | 12.56 ± 1.24 | 13.51 ± 0.99 | 13.01 ± 1.92 |
| **Macro-elements** | 528.33 ± 0.83 | 488.29 ± 46.23 | 539.21 ± 14.5 | 506.64 ± 26.84 | 527.72 ± 17.21 | 536.21 ± 1.02 | 527.99 ± 35.24 | 504.65 ± 56.07 |

**Table S3** continued.

| **Minerals** | **P1a** | **P2a** | **P2b** | **P3a** | **P3b** | **P4a** | **P4b** |
| --- | --- | --- | --- | --- | --- | --- | --- |
| Boron | 0.02 ± 0.01 | 0.01 | 0.03 | 0.01 | 0.01 ± 0.01 | 0.03 ± 0.02 | 0.03 ± 0.01 |
| Calcium | 1.55 ± 0.45 | 1.01 ± 0.61 | 1.56 ± 0.91 | 4.36 ± 8.06 | 5.56 ± 8.82 | 6.14 ± 9.37 | 0.99 ± 0.35 |
| Copper | 0.01 |  |  | 0.01 | 0.01 ± 0.01 | 0.01 |  |
| Iron | 0.08 ± 0.03 | 0.07 ± 0.01 | 0.07 ± 0.01 | 0.08 ± 0.02 | 0.06 ± 0.01 | 0.07 ± 0.03 | 0.10 ± 0.07 |
| Potassium | 6.86 ± 1.22 | 7.30 ± 0.46 | 7.88 ± 0.07 | 7.02 ± 0.83 | 6.55 ± 0.83 | 6.51 ± 1.32 | 5.36 ± 0.95 |
| Magnesium | 1.12 ± 0.23 | 1.20 ± 0.15 | 1.75 ± 0.75 | 1.34 ± 0.25 | 1.19 ± 0.27 | 1.11 ± 0.15 | 0.99 ± 0.05 |
| Manganese | 0.03 ± 0.01 | 0.03 | 0.02 ± 0.01 | 0.04 ± 0.03 | 0.05 ± 0.04 | 0.02 ± 0.01 | 0.03 ± 0.03 |
| Sodium | 0.25 ± 0.04 | 0.22 ± 0.05 | 0.21 ± 0.10 | 0.41 ± 0.43 | 0.25 ± 0.10 | 0.39 ± 0.47 | 0.18 ± 0.05 |
| Sulphur | 1.94 ± 0.35 | 2.06 ± 0.07 | 1.84 ± 0.66 | 1.70 ± 1.49 | 2.00 ± 0.34 | 2.22 ± 0.23 | 2.17 ± 0.31 |
| Zinc |  |  |  |  |  |  |  |
| **Phosphorus** | 4.92 ± 0.84 | 5.28 ± 0.31 | 5.85 ± 0.45 | 5.25 ± 0.51 | 4.80 ± 1.07 | 5.40 ± 0.94 | 4.47 ± 0.44 |
| **Nitrogen** | 32.87 ± 3.65 | 36.18 ± 2.32 | 30.03 ± 8.70 | 35.81 ± 3.26 | 35.02 ± 5.67 | 35.76 ± 3.42 | 35.87 ± 9.00 |
| **Carbon** | 478.39 ± 47.15 | 488.59 ± 3.16 | 473.34 ± 23.16 | 487.56 ± 16.39 | 481.31 ± 42.09 | 459.27 ± 55.20 | 497.5 ± 88.56 |
|  |  |  |  |  |  |  |  |
| Micro-elements | 11.57 ± 1.82 | 11.85 ± 0.67 | 13.24 ± 1.10 | 14.76 ± 6.27 | 15.56 ± 8.89 | 16.15 ± 10.41 | 9.60 ± 0.80 |
| **Macro-elements** | 516.18 ± 50.10 | 530.06 ± 4.27 | 509.22 ± 31.67 | 528.63 ± 18.90 | 521.14 ± 48.74 | 500.42 ± 57.14 | 537.84 ± 97.44 |

**Table S4.** Models best explaining the six recorded fitness parameters, highlighting the most influential explanatory variable(s) related to a) plant diversity (Biodiversity model: testing for effect of plant species richness (***pRi***), resource abundance and habitat type (plantation, forest, garden, ***H***) and b) food storage (Resource model: testing for effect of quantity (***rQt***) and nutritional quality). Variance of fixed effects (marginal *R²*: *_m_R²*) and variance of fixed and random effects (i.e. including effects of site and year; conditional *R²*: *_c_R²*) of the best models following AIC selection are shown, as are the AIC difference between the presented model and the NULL-model (Δ AIC). Asterisks indicate an interaction between fixed factors; ***NULL*** indicates that the NULL-model (i.e. random site and year effects only) best explained the observed variance in fitness. Note that the Resource model was only calculated when fitness and food resource parameters were simultaneously recorded (otherwise NA).

| Response variable | Biodiversity model | Δ AIC | *_m_R²* | *_c_R²* |  | Resource model | Δ AIC | *_m_R²* | *_c_R²* |
| --- | --- | --- | --- | --- | --- | --- | --- | --- | --- |
| Colony reproduction | ***pRi*** | 13.03 | 0.32 | NA |  | NA |  |  |  |
| Brood volume | ***pRi*** | 18.88 | 0.28 | 0.60 |  | ***rQt*** | 16.79 | 0.33 | 0.53 |
| Queen production | ***pRi*** | 4.81 | 0.12 | NA |  | ***rQt*** | 3.32 | 0.14 | NA |
| Worker production | ***pRi * H*** | 9.46 | 0.39 | 0.39 |  | ***NULL*** | - | - | - |
| Worker body fat | ***NULL*** | - | - | - |  | ***NULL*** | - | - | - |
| Worker body size | ***NULL*** | - | - | - |  | NA |  |  |  |

**SM 3. Selection of biodiversity variables tested**

Biodiversity-related explanatory variables were often correlated, including plant species richness and garden, forest and plantation area (see SM 1). To include only independent explanatory variables in all subsequent analyses, we restricted model comparison to models including plant species richness, resource abundance and habitat type (instead of area). To validate that plant species richness better explained variance than any of the correlated habitat areas, we tested all (covarying) biodiversity-related explanatory variables for colony reproduction in separate models (Table S5). Here, plant species richness clearly provided the highest explanatory power (over garden, forest and plantation area).

**Table S5.** Models testing the effect of single biodiversity-related explanatory variables on colony reproduction. Given are the AIC difference between the presented model and the NULL-model (Δ AIC), as well as the variance of the tested variable (marginal *R²*: *_m_R²*).

| Models | Δ AIC | *_m_R²* |
| --- | --- | --- |
| Plant species richness model | 13.03 | 0.32 |
| Garden area model | 5.28 | 0.24 |
| Forest area model | -0.51 | 0.05 |
| Plantation area model | 1.11 | 0.10 |
| Plant resource abundance model | -1.97 | <0.01 |

**References**

1 Michener, C. D. Observations on the nests and behavior of *Trigona* in Australia and New Guinea (Hymenoptera, Apidae). *American Museum Novitates* **2026**, 2-46 (1961).

2 Kaluza, B. F. *et al.* Generalist social bees maximize diversity intake in plant species-rich and resource-abundant environments. *Ecosphere* **8**, e01758 (2017).

3 Leonhardt, S. D. & Blüthgen, N. The same, but different: Pollen foraging in honeybee and bumblebee colonies. *Apidologie* **43**, 449-464 (2012).

4 Micheu, S., Crailsheim, K. & Leonhard, B. Importance of proline and other amino acids during honeybee flight. *Amino Acids* **18**, 157-175 (2000).

5 de Groot, A. P. Protein and amino acid requirements of the honey bee (*Apis mellifera* L.). *Physiologia Comparata et Oecologia* **3**, 197-285 (1953).

6 Ruedenauer, F. A., Spaethe, J. & Leonhardt, S. D. How to know which food is good for you: bumblebees use taste to discriminate between different concentrations of food differing in nutrient content. *J. Exp. Biol.* **218**, 2233-2240, doi:doi: 10.1242/jeb.118554 (2015).

7 Minden, V. & Kleyer, M. Internal and external regulation of plant organ stoichiometry. *Plant Biology* **16**, 897-907 (2014).

8 Murphy, J. & Riley, J. P. A modified single solution method for the determination of phosphate in natural waters. *Analytica Chimica Acta* **27**, 31-36 (1962).
